# Natural Language Processing Identification of Nonprescribed Fentanyl Use in Electronic Health Records: Algorithm Development and Validation Study

**DOI:** 10.2196/97485

**Published:** 2026-09-09

**Authors:** Joshua Trujeque, Joseph A Simonetti, Isai Ortiz, Nicholas E Ingraham, Nathan Mesfin, Jeremy Yeung, Rui Zhang, Lisa A Brenner, R Adams Dudley

**Affiliations:** 1 Minneapolis Veterans Affairs Healthcare System, Center for Care Delivery and Outcomes Research, One Veterans Drive, Minneapolis, MN, 55417, United States; 2 Rocky Mountain Mental Illness Research, Education, and Clinical Center for Suicide Prevention, Rocky Mountain Regional VA Medical Center, Aurora, CO, United States; 3 Injury and Violence Prevention Center, Colorado School of Public Health, University of Colorado Anschutz Medical Campus, Aurora, CO, United States; 4 Center for Veterans Research and Education, Minneapolis, MN, United States; 5 Northwell Health, New Hyde Park, NY, United States; 6 Department of Psychiatry, Zucker Hillside Hospital, Glen Oaks, NY, United States; 7 Division of Pulmonary, Allergy, and Critical Care Medicine, Department of Medicine, University of Minnesota Medical School, Minneapolis, MN, United States; 8 Institute for Health Informatics, University of Minnesota, Minneapolis, MN, United States; 9 Department of Surgery, University of Minnesota, Minneapolis, MN, United States; 10 Brain Health Coordinating Center, Rocky Mountain VA Regional Medical Center, Aurora, MN, United States; 11 Department of Physical Medicine and Rehabilitation, University of Colorado Anschutz Medical Campus, Aurora, CO, United States; 12 Department of Psychiatry, University of Colorado Anschutz Medical Campus, Aurora, CO, United States; 13 Department of Neurology, University of Colorado Anschutz Medical Campus, Aurora, CO, United States; 14 Center for Care Delivery and Outcomes Research, Minneapolis VA Health Care System, Minneapolis, MN, United States; 15 School of Public Health, University of Minnesota, Minneapolis, MN, United States

**Keywords:** fentanyl, natural language processing, opioid, overdose, veteran

## Abstract

**Background:**

Overdose and suicide due to nonprescribed fentanyl use have increased significantly, yet health care systems lack reliable methods to identify patients who use nonprescribed fentanyl. *International Classification of Diseases* codes are inconsistent and do not specify nonprescribed fentanyl use.

**Objective:**

This study aimed to develop natural language processing approaches to identifying nonprescribed fentanyl use in electronic health record (EHR) documentation.

**Methods:**

This retrospective study included Veterans Health Administration patients seen between April 5, 2023, and December 23, 2024. A term list was developed to identify fentanyl-related mentions in clinical text, and 250-character snippets surrounding identified mentions were extracted. Veterans (n=3878) were randomly sampled from 5 predefined groups based on the presence of 1 of 4 terms (“fent,” “blues,” “M30s,” and “tranq”) in their EHR documentation. Physician annotators classified snippets into “nonprescribed fentanyl use,” “prescribed fentanyl use,” or “other,” with interannotator agreement evaluated using the mean pairwise Cohen κ. Cross-validation folds were constructed at the patient level between training and test sets. Penalized logistic regression, Bio-ClinicalBERT, Llama 3-8B, and Mistral-7B were trained on labeled data and compared. Model performance was evaluated using precision, recall, and *F*_1_-scores for each class, with a focus on the nonprescribed fentanyl use class as the primary label of clinical interest using bootstrapped 95% CIs. A fairness analysis and Shapley additive explanations analysis were performed using Bio-ClinicalBERT. External validation was performed using Bio-ClinicalBERT on an independent sample of 200 snippets, each representing a unique patient from January 2025 to June 2026, with precision reported as the primary validation metric.

**Results:**

Of 7389 snippets, 9.6% (n=709) were classified as “nonprescribed fentanyl use,” 40.3% (n=2981) were classified as “prescribed fentanyl use,” and 50% (n=3699) were classified as “other.” Interannotator agreement was high (κ=0.822). Llama 3-8B achieved the highest *F*_1_-score for nonprescribed fentanyl use (0.87, 95% CI 0.83-0.92), followed by Mistral-7B (0.80, 95% CI 0.75-0.84), Bio-ClinicalBERT (0.80, 95% CI 0.74-0.85), and penalized logistic regression (0.74, 95% CI 0.73-0.75). Performance was consistent across demographic subgroups, with lower performance for the nonprescribed fentanyl use class observed in female and Hispanic subgroups. Shapley additive explanations analysis revealed clinically meaningful discriminating terms for each class, although subword tokens required contextual interpretation. External validation of Bio-ClinicalBERT demonstrated a precision of 0.79 for nonprescribed fentanyl use.

**Conclusions:**

Natural language processing can identify nonprescribed fentanyl use in EHR documentation, although model performance for this class was lower than overall model performance, reflecting the clinical complexity of identifying nonprescribed use and the variable ways in which clinicians document this problem. This approach may support risk prediction and targeting of interventions to patients exposed to nonprescribed fentanyl.

## Introduction

From 2003 to 2023, the United States age-adjusted drug overdose death rate increased from 8.9 to 31.3 per 100,000, largely attributed to nonprescribed use of fentanyl and fentanyl analogues, with 72,000 overdose deaths associated with nonprescribed fentanyl in 2023 [1,2]. Despite a recent reduction in US overdoses, nonprescribed fentanyl overdoses and related harms remain common across many populations [3,4].

Health care systems are critical intervention points for patients with nonprescribed fentanyl use. Patients who use nonprescribed fentanyl are at risk of developing opioid use disorder (OUD) and complications stemming from use, such as unintentional and intentional (eg, suicide) overdoses and skin wounds from adulterated drugs. The ability to identify patients who use nonprescribed fentanyl could enable health care systems and clinicians to identify opportunities to expand opioid-specific care, including counseling and initiation of medications for OUD. Furthermore, identifying individuals using nonprescribed fentanyl could support the development of system-level interventions focused on harm reduction, such as risk prediction models and distribution of naloxone and xylazine adulterant testing materials.

Efforts to address nonprescribed fentanyl use are hindered by the lack of reliable methods to identify patients. While there are *International Classification of Diseases* (*ICD*) codes for OUD, they are underused, do not distinguish between types of opioids used, and may not be applied by a clinician if they do not believe that a patient meets the criteria for OUD at that time (thus, information about opioid use is not captured in structured data) [5-7]. Discerning between patients who use fentanyl rather than other opioids is also important because risk of overdose and treatment strategies are dependent on the type of opioid used [8-10]. Understanding which specific substances (eg, fentanyl vs other opioids) contribute to opioid use, OUD, and related complications is also informative for policymakers. Furthermore, because fentanyl is commonly prescribed in health care settings, many text mentions reflect prescribed use (eg, procedural sedation), requiring scalable electronic health record (EHR) methods to distinguish between prescribed and nonprescribed use.

Natural language processing (NLP) is a potential solution to identify patients who use nonprescribed fentanyl. However, there is no published research describing NLP approaches to identify nonprescribed fentanyl use. Rather, NLP and other machine learning approaches have been developed to identify opioid overdose, misuse, and OUD for any kind of opioid rather than fentanyl specifically [11-14].

The Veterans Health Administration (VHA), the largest US health system with over 9 million enrollees, offers extensive EHR data for developing NLP methods to identify nonprescribed fentanyl use [15]. This is highly relevant to veterans as overdose mortality among them rose 53% from 2010 to 2019, with 93% of those deaths attributed to opioids [16]. In response, the VHA has developed and implemented a variety of interventions for OUD, including but not limited to distribution of naloxone and fentanyl testing strips and sterile syringe programs, as well as efforts to expand access to OUD counseling and medication treatment (eg, buprenorphine and methadone) [17-19]. Thus, clinicians may be prompted or encouraged to document fentanyl use across a variety of settings, and the texts they generate may be useful training data for building classifiers intended to identify a high-risk subset of patients with OUD. In addition, including data on nonprescribed fentanyl use may improve VHA overdose and suicide risk models [20]. Our aim was to describe the development and performance of different NLP approaches to identify nonprescribed fentanyl use through EHR data.

## Methods

We conducted a retrospective cross-sectional analysis of notes on patients who received VHA care from April 5, 2023, to December 23, 2024.

### Ethical Considerations

This study was approved by the Minneapolis VA Health Care System Institutional Review Board (number 1594855). We used the STROBE (Strengthening the Reporting of Observational Studies in Epidemiology) guidelines to describe study procedures and findings [21].

### Term Identification

We identified an initial list of 8 terms that clinicians might use to document fentanyl use through a combination of literature review; input from subject matter experts; and manual review of 100 EHR notes from patients with an *ICD*, *10th Revision* (*ICD-10*), code for OUD. These terms included “fentanyl,” “fent,” “blues,” “M30s,” “tranq,” “tranq dope,” “sleep dope,” and “fetty.” This list was narrowed down to 4 terms (“fent,” “tranq,” “M30s,” and “blues”) that appeared at least once in our sample.

### Study Population and Note Processing

We identified all patients whose EHR documentation included at least one mention of any fentanyl term. For note sampling, we stratified patient encounters a priori into 5 groups based on the expected context in which fentanyl was mentioned, such as addressing OUD (eg, during mental health encounters), prescribed use of fentanyl (eg, during a procedure), or other clinical scenarios. We selected these settings because we hypothesized that terms and documentation styles may differ across these settings. Groups included (1) OUD-related encounters (patients with notes that included a fentanyl term and whose notes were associated with a clinical encounter with an *ICD-10* code for OUD), (2) suicide or overdose event encounters (patients with a Suicide Behavior and Overdose Report [SBOR; a report created by VHA staff for all patients identified as having a suicide or overdose event] in which a fentanyl term was present in any note on or up to 90 days before the most recent SBOR entry), (3) non-OUD and noncrisis event clinical care setting encounters (patients with no SBOR or *ICD-10* code for OUD who nonetheless had at least one fentanyl term in an inpatient or outpatient encounter in the following care settings: primary care, mental health, inpatient medicine, medical intensive care unit, inpatient surgery, social work, chronic pain and wellness, or homeless care team), (4) periprocedure encounters (patients with a Current Procedural Terminology code for 1 of the 10 most frequent procedures performed at the VHA [eg, tissue exam by a pathologist] within 24 hours of a fentanyl term mention), and (5) a bias mitigation sample (a random sample of patients’ notes from any encounter or note type with a fentanyl term to address potential sources of bias). We initially sampled approximately 200 notes per group randomly and without replacement. Because the low prevalence of the target class limited performance, we added 1000 notes per group to improve model performance. For each identified fentanyl term, we extracted a 250-character snippet of surrounding text, a text length selected based on prior work [22] demonstrating sufficient context for classification while maintaining computational efficiency. These snippets were then labeled using a Microsoft Azure machine learning text data labeling project [23].

### Fentanyl Classification

We developed 3 classification labels for snippets containing fentanyl mentions: “nonprescribed fentanyl use,” “prescribed fentanyl use,” and “other.” “Nonprescribed fentanyl use” included mentions of fentanyl obtained and used without a prescription both currently and in the past. “Prescribed fentanyl use” included mentions of fentanyl that was prescribed for medical indications, including procedural sedation and transdermal pain treatment. The “other” category captured fentanyl mentions that did not meet the criteria for prescribed or nonprescribed use. This included concepts such as unspecified OUD; misuse; potential accidental exposure to fentanyl when consuming other nonprescribed drugs (eg, methamphetamines laced with fentanyl); fentanyl testing; fentanyl allergy documentation; uncertain or not enough context; negation of fentanyl use; fentanyl use by another person; standardized or templated clinical language about fentanyl, such as education materials about fentanyl risk that might be addended to a clinical note; and terms with alternative meanings unrelated to fentanyl use (eg, “blues” documented to describe depression). These subcategories were combined for modeling development because our primary objective was to identify individuals whose snippets contained reasonable clinical documentation of nonprescribed fentanyl use. Prior to annotation, a group of 6 physician annotators with experience treating patients with prescribed and nonprescribed fentanyl use created a conceptual classification schema and refined it over several sessions, in which annotators independently reviewed snippets and then met as a group to align on classification, developing consensus regarding the classification rules and definitions for each class. During annotation, each snippet was classified independently by at least 3 physicians prior to reaching a consensus classification or determining the need for further review. Interannotator agreement was assessed among the 6 physician annotators on approximately 30% of the snippets before consensus using mean pairwise unweighted Cohen κ values [24]. For snippets where annotators did not reach consensus, classification was determined through group discussion to prioritize precision over recall to minimize false positives, recognizing that in a clinical application, false positives could lead to unnecessary interventions. Snippets with consensus were used as the final dataset for training, validation, and testing of the NLP models.

### Development of NLP Models

On the basis of model performance in prior work [22], we reused the same nonneural and neural models and applied the same modeling framework: ridge-penalized logistic regression and Bio-ClinicalBERT. We also compared these 2 previously useful models to 2 other large language models (Mistral-7B [Mistral AI] and Llama 3-8B [Meta AI]) using instruction tuning, 10-fold cross-validation with 90% of data used for training and 10% held out for testing, and fixed random seeds to choose the initial value for parameters in each model to ensure reproducibility [25,26]. Cross-validation folds were split at the patient level to prevent overlap between training and test sets.

Model performance was evaluated using precision, recall, and *F*_1_-scores for each class. To estimate the uncertainty in model performance, the test set was bootstrapped 100 times by resampling snippets with replacement. Performance metrics were calculated for each bootstrap sample, and the mean and corresponding 95% CIs were reported. To interpret model predictions, we implemented Shapley additive explanations (SHAP) [27]. The highest overall *F*_1_-score from all folds was used to calculate SHAP values for the Bio-ClinicalBERT model. To address algorithmic bias, particularly in relation to demographic differences, a fairness analysis was performed on the Bio-ClinicalBERT model by stratifying each bootstrapped test set according to patient demographic characteristics, including sex (male or female), race (White or non-White), and ethnicity (Hispanic or non-Hispanic). Performance metrics were calculated separately for each subgroup, and the mean and 95% CIs were reported across the 100 bootstrap samples.

### External Validation

External validation was conducted to assess the real-world performance on identifying nonprescribed fentanyl use using data that were both temporally and patient independent from the original training and test sets. Using the same term-based snippet extraction method, we identified fentanyl mentions among VHA patients from January 2025 to June 2026, excluding all patients included in the original dataset. The fine-tuned Bio-ClinicalBERT model classified the extracted snippets, and 200 snippets classified as nonprescribed fentanyl use were randomly sampled, retaining the first snippet from the first note per patient so that each snippet referred to a unique patient to translate snippets into a patient-level identification. Each snippet was annotated using the same classification criteria. As identifying nonprescribed fentanyl use was the primary clinical objective, only model-positive cases were reviewed. Thus, only precision was calculated based on the proportion of confirmed patients upon manual review.

## Results

The demographics of the study sample are shown in Table 1. A total of 3878 patients were identified, including 847 (21.8%) in group 1, 908 (23.4%) in group 2, 678 (17.5%) in group 3, 784 (20.2%) in group 4, and 661 (17%) in group 5. The mean age, sex, race, and ethnicity of the 5 groups were comparable to those of the general VHA population [28]. Among the 7389 annotated snippets, the most common classification was “other” (n=3699, 50%), followed by “prescribed fentanyl use” (n=2981, 40.3%) and “nonprescribed fentanyl use” (n=709, 9.6%; Table 1). The most common term used to identify snippets was “fent” ( 7310/7403, 98.7%), followed by “blues” (176/7403%), “tranq” (14/7403, 0.2%), and “M30s” (0% 3/7403), indicating that these alternative terms to “fent” contributed minimally to overall yield. For all 15 pairwise physician annotator combinations, the mean pairwise κ was 0.822 (SD 0.074), indicating high agreement.

Following patient-level splitting, the training and test sets comprised 3610 and 881 unique patients, respectively, with no overlap. Table 2 shows model performance for classifying snippets as “nonprescribed fentanyl use,” “prescribed fentanyl use,” or “other.”

**Table 1.** Patient demographics and snippet classification by annotators for each patient group.

Demographics	Group 1 (*ICD-10*^a^ code for OUD^b^ and fentanyl term)	Group 2 (SBOR^c^ in previous year and fentanyl term)	Group 3 (various encounter types without *ICD-10* codes for OUD or SBOR and fentanyl term)	Group 4 (encounter with top 10 CPT^d^ codes within 24 h of a fentanyl term)	Group 5 (random sample of notes and fentanyl term)
Patients (n=3878), n (%)	847 (21.8)	908 (23.4)	678 (17.5)	784 (20.2)	661 (17)
Age (y), mean (SD)	56 (14.4)	54 (15.9)	63 (15.5)	60 (14.7)	60 (15.9)
Sex, n/N (%)					
Female	67/847 (7.9)	135/908 (14.9)	72/678 (10.6)	101/784 (12.9)	93/661 (14.1)
Male	780/847 (92.1)	773/908 (85.1)	606/678 (89.4)	683/784 (87.1)	568/661 (85.9)
Race, n/N (%)					
American Indian	6/847 (0.7)	11/908 (1.2)	5/678 (0.7)	8/784 (1)	3/661 (0.5)
Asian	4/847 (0.5)	12/908 (1.3)	4/678 (0.6)	8/784 (1)	7/661 (1.1)
Black	169/847 (20)	222/908 (24.4)	110/678 (16.2)	166/784 (21.2)	124/661 (18.8)
Hawaiian	14/847 (1.7)	7/908 (0.8)	7/678 (1.0)	11/784 (1.4)	12/661 (1.8)
Multiracial	5/847 (0.6)	10/908 (1.1)	5/678 (0.7)	9/784 (1.1)	7/661 (1.1)
Unknown	83/847 (9.8)	91/908 (10)	59/678 (8.7)	74/784 (9.4)	70/661 (10.6)
White	567/847 (66.9)	555/908 (61.1)	488/678 (72)	508/784 (64.8)	438/661 (66.3)
Ethnicity, n/N (%)					
Hispanic	51/847 (6)	86/908 (9.5)	65/678 (9.6)	69/784 (8.8)	69/661 (10.4)
Not Hispanic	729/847 (86.1)	748/908 (82.4)	574/678 (84.7)	661/784 (84.3)	550/661 (83.2)
Unknown	68/847 (8)	74/908 (8.1)	39/678 (5.8)	54/784 (6.9)	42/661 (6.4)
Fentanyl term prevalence, n (%)					
Notes (n=4496)	1092 (24.3)	996 (22.2)	910 (20.2)	832 (18.5)	666 (14.8)
Snippets (n=7389)^e^	2153 (29.1)	1493 (20.2)	1467 (19.9)	1265 (17.1)	1011 (13.7)
Snippet classification, n/N (%)					
Nonprescribed	493/2153 (22.9)	182/1493 (12.2)	12/1467 (0.8)	7/1265 (0.6)	15/1011 (1.5)
Prescribed	506/2153 (23.5)	372/1493 (24.9)	880/1467 (60)	685/1265 (54.2)	538/1011 (53.2)
Other	1154/2153 (53.6)	939/1493 (62.9)	575/1467 (39.2)	573/1265 (45.3)	458/1011 (45.3)

a *ICD-10*: *International Classification of Diseases, 10th Revision*.

b OUD: opioid use disorder.

c SBOR: Suicide Behavior and Overdose Report.

d CPT: Current Procedural Terminology.

e Counts represent the number of generated snippets that included a fentanyl-related term. Snippets may include more than one term.

**Table 2.** Comparison of model performance for classifying snippets as “nonprescribed fentanyl use,” “prescribed fentanyl use,” and “other.”

Models	Nonprescribed			Prescribed			Other		
	*F*_1_-score (95% CI)	Precision (95% CI)	Recall (95% CI)	*F*_1_-score (95% CI)	Precision (95% CI)	Recall (95% CI)	*F*_1_-score (95% CI)	Precision (95% CI)	Recall (95% CI)
Llama 3-8B	0.87 (0.83‐0.92)	0.88 (0.82‐0.93)	0.87 (0.80‐0.93)	0.96 (0.95‐0.97)	0.99 (0.98‐1.00)	0.94 (0.91‐0.95)	0.95 (0.94‐0.96)	0.93 (0.91‐0.95)	0.97 (0.96‐0.98)
Mistral-7B	0.80 (0.75‐0.84)	0.90 (0.82‐0.96)	0.72 (0.65‐0.79)	0.96 (0.95‐0.97)	0.99 (0.99‐1.00)	0.93 (0.92‐0.95)	0.95 (0.94‐0.96)	0.92 (0.90‐0.93)	0.98 (0.97‐0.99)
Bio-ClinicalBERT	0.80 (0.74‐0.85)	0.80 (0.72‐0.85)	0.80 (0.72‐0.89)	0.97 (0.96‐0.98)	0.97 (0.96‐0.98)	0.97 (0.96‐0.98)	0.94 (0.93‐0.96)	0.94 (0.93‐0.97)	0.94 (0.92‐0.97)
Penalized logistic regression	0.74 (0.73‐0.75)	0.75 (0.74‐0.76)	0.73 (0.72‐0.74)	0.97 (0.97‐0.97)	0.96 (0.96‐0.97)	0.97 (0.97‐0.97)	0.93 (0.93‐0.94)	0.94 (0.93‐0.94)	0.93 (0.93‐0.94)

Among the 4 models, the Llama 3-8B model demonstrated the best performance for nonprescribed fentanyl use (*F*_1_-score=0.87, 95% CI 0.83-0.92), followed by Mistral-7B (*F*_1_-score=0.80, 95% CI 0.75-0.84), Bio-ClinicalBERT (*F*_1_-score=0.80, 95% CI 0.74-0.85), and penalized logistic regression (*F*_1_-score=0.74, 95% CI 0.73-0.75). The CIs for Llama 3-8B and penalized logistic regression did not overlap, suggesting a meaningful performance difference. The CIs for Llama 3-8B, Mistral-7B, and Bio-ClinicalBERT overlapped substantially.

**Figure 1.**
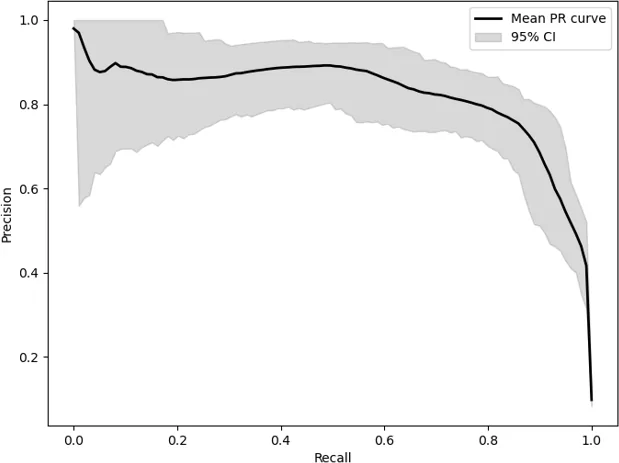
Bio-ClinicalBERT precision-recall (PR) curve for nonprescribed fentanyl use.

The precision-recall curve (Figure 1) for the nonprescribed fentanyl use class showed mean precision remaining above 0.80 across recall values up to approximately 0.6, after which precision declined as recall approached 1.0.

**Figure 2.**
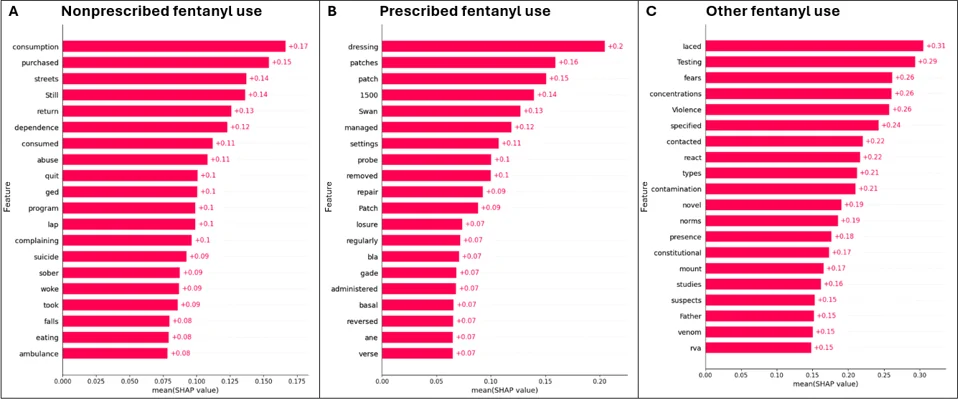
The top 20 features that include words or phrases most positively associated with each predicted classification based on the mean absolute Shapley additive explanations (SHAP) values from the Bio-ClinicalBERT model. Positive SHAP values are indicative of the predicted classification by the model.

The top 3 features (Figure 2) in the “nonprescribed fentanyl use” classification were “consumption,” “purchased,” and “streets.” The top 3 features in the “prescribed fentanyl use” classification were “dressing,” “patch(es),” and “1500,” and the top 3 features in the “other” classification were “laced,” “testing,” and “fears.” Table 3 shows additional features positively associated with each predicted classification in example snippets in various clinical contexts.

**Table 3.** Example snippets with features positively correlated with model classifications. Example snippets were adapted from a force plot of Shapley additive explanations [27] values from the Bio-ClinicalBERT model depicting the approximate majority of features that accounted for the total prediction value of each fentanyl classification.

Fentanyl classification and example context	250-character fentanyl snippet^a^
Nonprescribed fentanyl use	
Current use	“patient engagement – Other conditions contributing to risk: history of TBI, opioid dependence (street fentanyl 10 pills/d), tobacco use disorder (smokes tobacco and vapes), PTSD, obesity, OSA – UDS results reviewed”
Current use	“enies, quit ~6 months ago -Illicit substances: Denies any illicit substances, or prescription drug misuse/abuse. Last used fentanyl ~24H prior to Suboxone induction SUICIDE ROS: Veteran denied any suicide attempts or self-injurious behaviors recently.”
Historical use	“ndence, tobacco dependence, and cannabis dependence. Veteran admitted many years ago for accidental drug overdose (fentanyl) with hypoxia. Veteran shortly after received vivitrol monthly injection with success of abstinence from opioid use.”
Prescribed fentanyl use	
Procedural sedation	“ny adjunctive medications given during procedure and any reversal agents required. time:11:29 midazolam: 2 mg iv push fentanyl: 75 mcg iv push vital signs monitored and recorded every 5 minutes: time: bp: hr: rr: spo2 %: 11:34”
Medication list	“xime proxetil 200mg tab 200mg 1 tablet oral bid Diclofenac na 1% top gel 4 gm (dosing card line) 4.5 inches topical qid Fentanyl tts--75mcg/hr patch 1 patch transdermal q48h Fluconazole 200mg tab 200mg 1 tablet oral qday Lidoca-alum/mag hydrox sus”
Inpatient administration	“infusion dopamine epinephrine (adrenalin) 2,000 mcg in sodium chloride 0.9 % 250 ml infusion 30 mcg/min (11/02/23 0125) fentanyl fentanyl lactated ringers 100 ml/hr at 11/02/23 0315 lidocaine norepinephrine 30 mcg/min (11/02/23 0015) phenylephrine”
Other	
Drug testing	“OXYCODONE Positive ng/mL Ref: NEGATIVE <100 ng/mL ***Reminder: Several opioids including fentanyl, methadone, buprenorphine, and tramadol are not included in the standard Urine Drug Screen testing. Please order”
Possible exposure due to other drug	“with RUL area of consolidation significant for pneumonia. Suspect patient could have possibly used cocaine (laced with fentanyl?) and passed out, leading to possible aspiration event given location of infiltrate. Patient normotensive and mildly tac”
Allergy	“allergy/adr -------- ----------- name name fentanyl name name morphine name name oxybutynin chloride iron mountai”

a Some snippets were altered if they contained identifying information.

Model performance was similar across demographic subgroups for the “prescribed fentanyl use” and “other” classes (Table 4). For the “nonprescribed fentanyl use” class, *F*_1_-scores were comparable across demographic subgroups except in the female subgroup, which showed wider CIs (0.76, 95% CI 0.43-0.94) than the male subgroup (0.8, 95% CI 0.75-0.85). Similarly, the Hispanic subgroup demonstrated lower performance (*F*_1_-score=0.71, 95% CI 0.50-0.84) than the non-Hispanic subgroup (*F*_1_-score=0.82, 95% CI 0.77-0.86). External validation of Bio-ClinicalBERT on an independent sample of 200 snippets, each representing a unique patient, from January 2025 to June 2026 demonstrated a precision of 0.79 (158/200, 79%), consistent with test set precision (0.80, 95% CI 0.72-0.85).

**Table 4.** Fairness analysis of model performance for nonprescribed fentanyl use.

Subgroup	Patients, n	Nonprescribed, *F*_1_-score (95% CI)	Prescribed, *F*_1_-score (95% CI)	Other, *F*_1_-score (95% CI)
Overall	1482	0.80 (0.74‐0.85)	0.97 (0.96‐0.98)	0.94 (0.93‐0.96)
Sex				
Male	1344	0.80 (0.75‐0.85)	0.97 (0.96‐0.98)	0.94 (0.92‐0.95)
Female	138	0.76 (0.43‐0.94)	0.98 (0.96‐1.00)	0.97 (0.94‐0.99)
Race				
White	943	0.82 (0.75‐0.87)	0.96 (0.95‐0.98)	0.94 (0.93‐0.96)
Non-White	539	0.78 (0.68‐0.87)	0.98 (0.97‐0.99)	0.94 (0.92‐0.96)
Ethnicity				
Hispanic	231	0.71 (0.50‐0.84)	0.99 (0.98‐1.00)	0.94 (0.90‐0.97)
Non-Hispanic	1251	0.82 (0.77‐0.86)	0.97 (0.96‐0.98)	0.94 (0.93‐0.96)

## Discussion

### Principal Findings

Despite recent declines in US overdose mortality, use of nonprescribed fentanyl remains a major driver of unintentional and intentional (eg, suicide) overdose deaths. An important challenge impeding clinical and research efforts is that health care systems lack methods to identify patients who use nonprescribed fentanyl. Using VHA clinical notes, we developed and validated NLP models to classify mentions as “nonprescribed use,” “prescribed use,” and “other.” Our fine-tuned models demonstrated strong performance, achieving *F*_1_-scores above 0.8, indicating that all models tested in this study were largely comparable overall.

Although Llama 3-8B achieved the highest nonprescribed fentanyl use *F*_1_-score (0.87), the substantially overlapping CIs among Llama 3-8B, Mistral-7B, and Bio-ClinicalBERT suggest comparable performance among these models, whereas the nonoverlapping CIs between Llama 3-8B and penalized logistic regression suggest a significant performance difference. Although external validation demonstrated comparable precision for identifying nonprescribed fentanyl use, this finding should be interpreted as preliminary because it did not validate full model performance. Each approach comes with trade-offs in terms of computation effort and cost, ability to identify patients across the health care system, and the certainty with which automated identification is truly indicative of nonprescribed fentanyl use. Our findings suggest that health systems with limited computational infrastructure or programming staff could reasonably implement penalized logistic regression or Bio-ClinicalBERT, which may provide improved identification compared with approaches relying solely on *ICD-10* codes [29]. We used Bio-ClinicalBERT for supplementary analyses given its lower computational cost and comparable performance to that of larger large language models, making it pragmatic for real-world deployment. Conversely, if a health system aimed to conduct targeted outreach for patients with nonprescribed fentanyl use but lacked resources to contact all identified patients, choosing a model with higher precision (positive predictive value) may be preferred to maximize the probability that contacted patients truly have nonprescribed fentanyl use. In this scenario, Mistral-7B may be favored despite its lower *F*_1_-score for nonprescribed fentanyl use (Table 2) as its reduced *F*_1_-score performance was due to lower recall (0.72), whereas its precision was higher than that of Bio-ClinicalBERT or Llama 3-8B. However, this trade-off deserves careful consideration because potentially missing 28% of patients with non-prescribed fentanyl use may be detrimental to clinical applications aimed at identifying and treating at-risk patients. In these contexts, a missed identification represents a missed clinical opportunity to prevent the harms associated with nonprescribed fentanyl use.

Overall, model performance was comparable across subgroups, with the exception of female and Hispanic subgroups for the “nonprescribed fentanyl use” class, where performance was somewhat lower. This finding is likely due to the smaller sample sizes among these groups, as suggested by the wider CIs (n=138 female vs n=1344 male; n=231 Hispanic vs n=1251 non-Hispanic). These findings should be interpreted as preliminary given the small subgroup sample sizes and wide CIs and warrants further investigation in larger samples. Future work should explore approaches to improve performance in underrepresented groups, such as oversampling these subgroups during training or subgroup-specific model fine-tuning.

The “other” category was the most common classification, accounting for half (3699/7389, 50%) of the annotated snippets, highlighting the diverse ways in which fentanyl is referenced in clinical text. This heterogeneity included drug testing results, allergy documentation, fentanyl use by another person, and often ambiguous fentanyl mentions. A notable subset included incidental or unintentional exposure to nonprescribed fentanyl through the intentional use of other substances such as methamphetamine or cocaine that were laced with fentanyl, which highlights the clinical complexity of this domain. Although consolidating these subcategories into a single “other” label may bias the model by obscuring clinically relevant distinctions, such as unintentional exposure and diagnostic uncertainty, this decision was made to avoid additional class imbalance that would have precluded model convergence. Future work could explore the classification of these clinically relevant concepts.

The methods we describe could be applied to support clinical, operational, and research efforts to reduce the harms associated with nonprescribed fentanyl use among VHA patients. Our approach could also be adapted in other health systems to identify high-risk patients and inform the targeting of other interventions. For example, this information could be incorporated into risk prediction models for overdose and suicide risk [30-32]. Identifying patients at risk could also facilitate large-scale efforts in harm reduction, such as prescribing naloxone and xylazine test distribution. Additionally, health care systems could use this approach for epidemiologic surveillance of fentanyl use, identification of at-risk patients who might not be receiving appropriate care, or evaluations of clinical care processes and outcomes among this patient population. These applications may require the use of evaluation frameworks other than standard aggregate model performance reporting to ensure clinical reliability across patient populations [33]. The evaluation approach in this study reporting class-specific performance and fairness analysis reflects this standard. Additionally, these clinical tools may require more evaluation prior to integration into clinical decision-making systems, such as prospective evaluation, cost-benefit analysis, and further consideration of the risks associated with misclassification.

Aside from the potency of fentanyl, some of the mortality and negative outcomes associated with fentanyl at the population level are likely attributable to its dramatic increase in use in such a short time (and health care systems’ lack of methods to identify and treat vulnerable patients). Importantly, this study provides a blueprint to develop automated methods that can help in identifying use of other potentially emerging substances, such as use of nitazenes and other newly developed synthetic opioids.

Our findings also highlight opportunities for further research with important clinical and operational implications. All approaches we used demonstrated lower performance for identifying nonprescribed fentanyl use than for identifying the “prescribed use” and “other” categories. On the basis of review of thousands of snippets, and as reflected in Table 3, those differences are due to greater linguistic variability in how nonprescribed fentanyl use is documented in clinical notes. Prescribed use is usually documented with dosages and often in a list of other medications. Common examples of the “other” category include templated drug testing results or documentation in allergy lists. In contrast, documentation indicating actual or potential nonprescribed use is heterogeneous and may include contextual references that challenge model interpretation, such as drug use by family members, nonprescribed use of other substances, or historical fentanyl use mentioned alongside currently active use disorders for other substances such as alcohol. Although these scenarios were categorized as “other” during annotation to create gold-standard training data, their semantic similarity to nonprescribed use plausibly contributed to misclassification. Future research could address developing models to better recognize these complexities.

Our specific findings—such as that Mistral-7B had higher precision—may not be generalizable to non-VHA systems given differences in care delivery, EHR formats, documentation, and patient demographics between systems. However, these findings are potentially impactful given that the VHA provides care to nearly 10 million veterans. Furthermore, our general approach to identifying fentanyl terms, extracting clinical documentation among relevant patient groups, annotation, and training and validating models could be applied in other health care settings.

The SHAP-associated terms should be interpreted cautiously because several high-ranking features were subword tokens generated by the Bio-ClinicalBERT tokenizer rather than stand-alone clinical concepts. Manual review showed that some fragments were mapped to clinically meaningful parent terms when considered in context. For example, in the prescribed fentanyl use classifier, “verse” appeared as part of “versed” or midazolam, “ane” appeared as part of “anesthesia,” “bla” appeared as part of “ablation,” “losure” appeared as part of “closure,” and “gade” appeared as part of “Tegaderm,” all of which are plausibly associated with procedural settings where fentanyl may be prescribed or administered. However, these subword attributions should not be interpreted independently of their parent terms and surrounding note context. Therefore, SHAP analyses were used as supportive model inspection tools rather than definitive explanations of model behavior.

This study has other limitations. It relied on information documented in clinical notes, and the accuracy or completeness of these records is unknown. Additionally, the sampling strategy relied on predefined fentanyl terms, and while this term list was developed systematically, no new terms were identified during the annotation process. However, this approach may miss implicit mentions or emerging slang terms not captured at the time of development and could impact the sensitivity of a full identification pipeline in a real-world setting.

### Conclusions

Identifying patients with nonprescribed fentanyl use is essential for patient care and informing public policy and system-level prevention and treatment strategies. As *ICD-10* codes have low sensitivity for nonprescribed OUD and no codes are specific to patients with nonprescribed fentanyl use, extracting this information from EHR documentation is a crucial step in mitigating the harms of fentanyl use. We describe models with strong performance in identifying nonprescribed fentanyl use from clinical text, offering a potential tool for improving detection and intervention efforts.

## Abbreviations

EHR: electronic health record
ICD: International Classification of Diseases
ICD-10: International Classification of Diseases, 10th Revision
NLP: natural language processing
OUD: opioid use disorder
SBOR: Suicide Behavior and Overdose Report
SHAP: Shapley additive explanations
STROBE: Strengthening the Reporting of Observational Studies in Epidemiology
VHA: Veterans Health Administration
